# Deep Vein Thrombosis in Critically Ill Patients With COVID‐19 Pneumonia: Incidence, Wells Score Diagnostic Performance, and Hospital Prognosis

**DOI:** 10.1002/jum.70046

**Published:** 2025-09-10

**Authors:** Clévio Cezar da Fonseca, Hugo Perazzo, Sandra Wagner Cardoso, Isabel Cristina Ferreira Tavares, Maria Pia Diniz Ribeiro, Rodrigo de Carvalho Moreira, Lara Esteves Coelho, Emília Moreira Jalil, André Miguel Japiassú, Elias Pimentel Gouvêa, Estevão Portela Nunes, Hugo Boechat Andrade, Luciano Barros Gouvêa, Marcel Treptow Ferreira, Pedro Mendes Azambuja‐Rodrigues, Ronaldo Ismerio Moreira, Kim Mattos Geraldo, Lucilene Araújo de Freitas, Vinicius Velleda Pacheco, Beatriz Gilda Jegerhorn Grinsztein, Pedro Emmanuel Alvarenga Americano do Brasil

**Affiliations:** ^1^ Evandro Chagas Infectious Diseases National Institute Oswaldo Cruz Foundation Rio de Janeiro Brazil

**Keywords:** COVID‐19, length of stay, mortality, ultrasonography, venous thrombosis

## Abstract

**Objectives:**

The risk of major venous thromboembolism (VTE) among patients with COVID‐19 is high but varies with disease severity. Estimate the incidence of lower extremity deep venous thrombosis (DVT) in critically ill hospitalized patients with COVID‐19, validate the Wells score for DVT diagnosis, and determine patients' prognosis.

**Methods:**

This was an observational follow‐up study in the context of the diagnosis and prognosis of DVT. All patients were hospitalized in the intensive care unit (ICU) of the Evandro Chagas National Institute of Infectious Diseases. Participants with COVID‐19 pneumonia were included. Lower‐limb Doppler to assess DVT was performed at admission and follow‐up. Prognosis outcomes were death, length of stay, need for mechanical ventilation, vasopressor use, and hemodialysis. Wells' score sensitivity and specificity were estimated at admission. Survival curves were estimated for patients with DVT and adjusted for the SAPS 3 score.

**Results:**

Between June 2020 and January 2021, 186 patients were included. The DVT incidence was 0.097. A Wells score of 2 or higher had a sensitivity and specificity of 1.00 and 0.94, respectively. Mortality and mechanical ventilation support were higher in participants with DVT. For these outcomes, after SAPS 3 adjustment, participants with DVT had twice the hazard of those without DVT. A web calculator (https://pedrobrasil.shinyapps.io/INDWELL/) is available for predictions.

**Conclusions:**

One can use the Wells score to accurately diagnose DVT in critically ill patients with COVID‐19. DVT increases the severity of COVID‐19, which highlights the importance of its early diagnosis, treatment, and prophylaxis in the ICU setting.

**Abbreviations**
CCI: Charlson Comorbidity Index
COVID‐19: coronavirus disease 2019
DVT: deep venous thrombosis
ECMO: extracorporeal membrane oxygenation
ICU: intensive care unit
IL: interleukin
IMV: invasive mechanical ventilation
INI/FIOCRUZ: Instituto Nacional de Infectologia Evandro Chagas
POCUS: point‐of‐care ultrasound
PTE: pulmonary thromboembolism
SAPS 3: Simplified Acute Physiology Score version 3
SARS‐CoV‐2: severe acute respiratory syndrome coronavirus 2
SBACV: Brazilian Society of Angiology and Vascular Surgery
SIC: sepsis‐induced coagulopathy
SOFA: sequential organ failure assessment
US: ultrasonography
VTE: venous thromboembolism
WHO: World Health Organization

By October 2022, 617,787,221 Coronavirus disease 2019 (COVID‐19) cases and 6,545,929 deaths due to COVID‐19 were confirmed worldwide. Brazil has recorded 34,678,510 cases and 686,254 deaths in the same period.^1^ Between 10.96% and 22.90% COVID‐19 cases may progress, requiring hospitalization in intensive care units (ICU).^2^, ^3^ The overall COVID‐19 mortality in critically ill patients is variable, ranging from 5.6%^3^ to 49%,^4^ with the length of stay ranging from a few days to several weeks.^5^ Additionally, the risk of mechanical ventilation requirement was estimated to be 7.1%.^3^

Severe acute respiratory syndrome coronavirus 2 (SARS‐CoV‐2) may directly cause endothelial injury. Deep vein thrombosis (DVT) and pulmonary thromboembolism (PTE) have been described as COVID‐19 clinical findings. However, the mechanism by which COVID‐19 increases the DVT and PTE risks is not fully understood.^6^ Nevertheless, features related to these clinical findings, such as coagulation disturbances, thrombocytopenia, elevated D‐dimer, and abnormalities in the clotting cascade, are often present and likely to be related to DVT and mortality.^4^

Studies with Italian and Spanish populations showed DVT incidences among critically ill COVID‐19 patients ranging from 7.7% to 46%.^2^, ^7^, ^8^, ^9^ Studies in Brazil demonstrated a DVT incidence ranging from 1.6 to 36.8% among critically ill COVID‐19 patients.^10^, ^11^, ^12^, ^13^ Additionally, DVT is substantially higher in critically ill patients with COVID‐19 compared to critically ill patients without COVID‐19, particularly in the ICU setting.^14^, ^15^

The risk of major venous thromboembolism (VTE) among patients with COVID‐19 is approximately 24%, and varies with disease severity, thromboprophylaxis protocols, and diagnostic strategies.^16^, ^17^, ^18^ In contrast, historical rates of VTE in non‐COVID‐19 ICU populations are generally lower, often in the range of 5–15% with standard thromboprophylaxis.^19^ Therefore, VTE in ICU patients with COVID‐19 can be 3 times the VTE in non‐COVID‐19 patients.^14^

DVT among COVID‐19 patients worsens the prognosis, increasing ICU admission, mechanical ventilation requirement,^20^ length of stay,^21^ and mortality.^22^ The mortality in critically ill patients with COVID‐19 with DVT ranges from 11.4 to 70.0%.^11^, ^13^, ^23^, ^24^ DVT is common among those already requiring mechanical ventilation, and the coexistence of DVT and respiratory failure is a hallmark of severe COVID‐19. The relationship is likely bidirectional: severe COVID‐19 increases the risk of both respiratory failure and DVT, and the presence of DVT may further complicate the clinical course.^12^, ^25^ In studies that performed routine ultrasonographic screening, DVT‐positive cohorts showed mechanical ventilation rates between 56.1 and 100%.^26^, ^27^, ^28^, ^29^ These prognosis rates do not seem to be different when estimated in the Brazilian population.^11^, ^26^, ^30^, ^31^, ^32^

In 1997, Wells^33^ developed a clinical score to conduct diagnostic investigations and classify patients according to their DVT risk. This score is currently used and recommended by guidelines^34^, ^35^ to estimate DVT diagnosis probability before performing ultrasonography (US).

Twenty percent of distal DVT will progress to proximal thrombosis, increasing the risk of death. Therefore, identifying the DVT location and classifying its type is crucial for determining the treatment strategies.^36^ The Brazilian Society of Angiology and Vascular Surgery (SBACV) Guidelines Project (2015) outlines detailed diagnostic and treatment recommendations for different DVT locations and classifications.^36^ However, studies regarding Wells' performance on diagnosing either VTE or PTE in COVID‐19 patients are scarce, have mixed hospitalized populations, and have heterogeneous results. Usually, the Wells score is combined with other biomarkers to improve diagnostic performance.^37^, ^38^, ^39^, ^40^, ^41^, ^42^, ^43^, ^44^, ^45^ We were not able to find any studies on this topic with the Brazilian population.

Efforts have been made to establish whether intensified thromboprophylaxis regimens are required for critically ill patients with COVID‐19. The best thromboprophylaxis should balance the risks of thrombosis and bleeding. The American Society of Hematology guidelines on thromboprophylaxis in patients with COVID‐19 stated the importance of an individualized decision for each patient based on an assessment of thrombosis and bleeding risk.^46^ Therefore, timely assessment of DVT and implementation of preventive strategies are necessary for a favorable prognosis of critically ill patients with COVID‐19.^47^

## Objectives

This study aims to estimate the DVT incidence in patients with COVID‐19 hospitalized in a critical care unit. This study also aims to validate the Wells score for DVT diagnosis among critically ill patients with COVID‐19 and to estimate the prognosis of patients with COVID‐19 and DVT.

## Methods

All participants or their legal representatives signed an informed consent form before enrolling in RECOVER‐SUS‐BRASIL. The Ethics Committee INI/FIOCRUZ approved the DVT sub‐study protocol on October 9, 2020, and can be found at https://plataformabrasil.saude.gov.br/visao/publico/indexPublico.jsf with the number CAEE 32449420.4.1001.5262. This report followed the STARD reporting guideline.

DVT diagnosis and prognosis in patients with COVID‐19 is a RECOVER‐SUS [NCT04807699] sub‐study. RECOVER‐SUS is a prospective observational multicenter follow‐up study of hospitalized patients with COVID‐19 performed in 7 public tertiary hospitals across 5 cities in Brazil.^48^ The DVT sub‐study was conducted at Instituto Nacional de Infectologia Evandro Chagas (INI/FIOCRUZ) only. INI/FIOCRUZ is part of the public health system. INI/FIOCRUZ is specialized in infectious diseases healthcare. At the time of the COVID‐19 pandemic, it had between 140 and 200 beds, including intermediate and ICU beds dedicated to COVID‐19 care. INI/FIOCRUZ admitted patients transferred from other health units, emergency rooms, from primary and intermediate complexity, including from neighboring cities.

Patients eligible for participation in the RECOVER‐SUS study included critically hospitalized patients, aged 18 years or older, with evidence of SARS‐CoV‐2 infection as per World Health Organization (WHO) criteria^49^ within 14 days of the onset of symptom initiation, confirmed through PCR testing of nasopharyngeal, oropharyngeal, or tracheal aspirate samples. These individuals also had chest imaging abnormalities at the time of screening. Participants without suspected, probable, or confirmed SARS‐CoV‐2 infection according to the WHO COVID‐19 guidelines were excluded.

This sub‐study included a cross‐sectional diagnostic study at the time of ICU admission and an observational follow‐up prognostic study developed in one of the ICUs (ICU‐H unit). During the study period, the ICU was exclusively for COVID‐19 patients. All patients hospitalized in ICU‐H during the inclusion period were sequentially screened for the sub‐study. In addition to the RECOVER‐SUS criteria, the sub‐study inclusion criteria included indication for ICU admission due to COVID‐19‐related respiratory complications, such as oxygen saturation ≤ 94% or the requirement for supplemental oxygen (including non‐invasive positive pressure ventilation or high flow supplemental oxygen), invasive mechanical ventilation (IMV), or extracorporeal membrane oxygenation (ECMO), and the performance of deep vein US in the first 48 hours of admission. The screening period for the DVT sub‐study was from June 2020 to January 2021. The DVT sub‐study has a retrospective part, checking and extracting data from June to October 2020, and a prospective part, going along with the original RECOVER‐SUS effort up to the end of the data collection period. Vaccination in Brazil began on January 17, 2021; therefore, all participants were not vaccinated against COVID‐19.^50^

Socio‐demographic characteristics, comorbidities, COVID‐19 symptoms, vital signs, and anthropometric measurements (weight and height) were recorded at hospital admission (baseline). Clinical data, blood samples, and ultrasound images for DVT verification (identifying the incidence and sites of location) were collected by trained investigators at baseline and on days 3, 7, 14, 21, and 28 of hospitalization. The study data were collected and managed using the REDCap electronic data capture tool hosted at INI/FIOCRUZ. All participants were monitored continuously from the time of hospital admission until they were either transferred to another institution, discharged, deceased, or until the sub‐study was interrupted.

The severity scores and Wells' score were obtained upon ICU admission independently of the ultrasound test and registered in the electronic medical charts. The Wells' score questions pertained to the incidence of active cancer treatment or palliation within 6 months, history of being bedridden recently either for more than 3 days or the occurrence of major surgery within 12 weeks; calf swelling in 1 leg measuring more than 3 cm in comparison to the other leg, with measurements taken 10 cm below the tibial tuberosity; the presence of collateral (non‐varicose) superficial veins; swelling in the entire leg; localized tenderness along the deep venous system; pitting edema, confined to the symptomatic leg; paralysis, paresis, or recent plaster immobilization of the lower extremity; previously documented DVT alternative diagnosis, as likely or more likely. The Wells score ranges from 2 to 9; the higher the score, the more likely the DVT diagnosis.^51^, ^52^ The Charlson Comorbidity Index (CCI),^53^ the sequential organ failure assessment (SOFA),^54^ the Simplified Acute Physiology Score version 3 (SAPS 3),^55^, ^56^, ^57^, ^58^ and the sepsis‐induced coagulopathy (SIC)^59^ are usually used to predict mortality either in general, or due to comorbidities, organ failure, or sepsis. These scores allow prognosis estimation adjustments.

Point‐of‐care US DVT was assessed by a critical care physician trained in point‐of‐care ultrasound (POCUS), independently of the Wells and severity scores. The protocol to perform the test was to begin at the common femoral vein and move distally, repeating the procedure on the greater saphenous vein, always compressing at 1–2 cm intervals until the vessel was no longer visible. The protocol included visualization of the entire proximal superficial femoral vein using a standard linear probe. Finally, the popliteal vein was compressed.^60^ The equipment available to perform POCUS DVT was Viamo™ c100, a portable US system of a cart‐based machine.^61^ For the validation of the Wells score, the US was used as the reference test. For prognostic purposes, the following outcomes were considered: overall death, length of stay, need for mechanical ventilation, vasopressor use, and hemodialysis.

The DVT sub‐study sample size was determined by the hospitalization capacity of the ICU during the predetermined period of inclusion, which at the time was believed to be between 180 and 250. Baseline clinical characteristics were tabulated to show either their frequencies or central tendencies according to their format. Regarding the diagnostic aspect, we estimated the sensitivity and specificity for the Wells' (and other) scores along with their 95% confidence intervals, estimated using a binomial distribution with the Wilson method, with the actual score being considered as a positive test result. For this analysis, DVT at US at any moment of hospitalization was the reference test. Regarding the prognostic aspect, the DVT US diagnosis was adjusted with the SAPS 3 score using either a Cox proportional hazards model or a linear model, depending on the prognosis being considered. The time‐dependent survival approach was used to consider participants who developed DVT either at baseline or during the follow‐up period. The Wald test at 5% was used for significance.

## Results

During the study period, 229 participants were screened. Of these, 43 were excluded because they were not subjected to the US within the first 48 h. A total of 186 patients were included in this analysis. DVT occurred in 18 participants; consequently, the incidence and its respective 95% confidence interval were determined to be 0.097 [0.062–0.148]. Of these, 10 participants were diagnosed with DVT within the first 24 hours of admission, 1 participant was diagnosed after 3 days, while 6 participants were diagnosed after 7 days, and 1 patient was diagnosed after 21 days of admission.

Age, sex, and ethnicity were similar between the participants within the DVT groups. Additionally, there were no evident differences in the DVT groups concerning the history of exposure, duration of symptoms, or physiological variables (Table 1). Pre‐existing comorbidities that were more frequent among the participants included hypertension, diabetes, and obesity. All 3 comorbidities occurred more frequently among participants without DVT (Table 1).

**Table 1:** Clinical, Demographic, Comorbidities, and Severity Scores Baseline Data by Deep Venous Thrombosis Diagnosis

	Deep Venous Thrombosis		
	No (n = 168)	Yes (n = 18)	Overall (n = 186)
Age (years)			
Mean (SD)	62.5 (14.6)	66.7 (10.6)	62.9 (14.3)
Median [Min, Max]	64.0 [22.0, 90.0]	67.5 [52.0, 90.0]	65.0 [22.0, 90.0]
Age (years)			
[20,30]	4 (2.4%)	0 (0%)	4 (2.2%)
(30,40]	11 (6.5%)	0 (0%)	11 (5.9%)
(40,50]	19 (11.3%)	0 (0%)	19 (10.2%)
(50,60]	35 (20.8%)	5 (27.8%)	40 (21.5%)
(60,70]	41 (24.4%)	7 (38.9%)	48 (25.8%)
(70,80]	42 (25.0%)	5 (27.8%)	47 (25.3%)
(80,90]	16 (9.5%)	1 (5.6%)	17 (9.1%)
Sex at birth			
Male	89 (53.0%)	11 (61.1%)	100 (53.8%)
Female	79 (47.0%)	7 (38.9%)	86 (46.2%)
Ethnicity			
White	35 (20.8%)	3 (16.7%)	38 (20.4%)
Black	12 (7.1%)	2 (11.1%)	14 (7.5%)
Mixed	114 (67.9%)	13 (72.2%)	127 (68.3%)
Asian	0 (0%)	0 (0%)	0 (0%)
Native American	0 (0%)	0 (0%)	0 (0%)
Missing	7 (4.2%)	0 (0%)	7 (3.8%)
Same household COVID‐19			
Yes	41 (24.4%)	5 (27.8%)	46 (24.7%)
No	83 (49.4%)	12 (66.7%)	95 (51.1%)
Missing	44 (26.2%)	1 (5.6%)	45 (24.2%)
Length of symptoms (days)			
Mean (SD)	8.99 (8.82)	9.53 (13.5)	9.04 (9.30)
Median [Min, Max]	7.00 [0, 86.0]	6.00 [2.00, 60.0]	7.00 [0, 86.0]
Missing	0 (0%)	1 (5.6%)	1 (0.5%)
Systolic blood pressure (mmHg)			
Mean (SD)	142 (25.2)	157 (31.4)	144 (26.1)
Median [Min, Max]	140 [70.0, 211]	150 [110, 240]	141 [70.0, 240]
Missing	1 (0.6%)	2 (11.1%)	3 (1.6%)
Diastolic blood pressure (mmHg)			
Mean (SD)	83.0 (14.3)	89.1 (19.3)	83.6 (14.8)
Median [Min, Max]	82.0 [40.0, 120]	89.0 [60.0, 129]	83.0 [40.0, 129]
Missing	1 (0.6%)	2 (11.1%)	3 (1.6%)
Heart beat (bpm)			
Mean (SD)	90.1 (15.5)	100 (19.0)	90.9 (16.0)
Median [Min, Max]	90.0 [44.0, 134]	101 [73.0, 130]	90.0 [44.0, 134]
Missing	1 (0.6%)	2 (11.1%)	3 (1.6%)
Respiratory frequency (bpm)			
Mean (SD)	24.8 (6.29)	26.2 (6.56)	24.9 (6.30)
Median [Min, Max]	24.0 [12.0, 45.0]	25.0 [18.0, 38.0]	24.0 [12.0, 45.0]
Missing	2 (1.2%)	3 (16.7%)	5 (2.7%)
Pulse oximetry (%)			
Mean (SD)	91.3 (8.36)	88.8 (10.0)	91.1 (8.53)
Median [Min, Max]	94.0 [53.0, 100]	92.5 [63.0, 98.0]	94.0 [53.0, 100]
Missing	11 (6.5%)	2 (11.1%)	13 (7.0%)
Hypertension			
No	58 (34.5%)	8 (44.4%)	66 (35.5%)
Yes	110 (65.5%)	10 (55.6%)	120 (64.5%)
Diabetes			
No	100 (59.5%)	12 (66.7%)	112 (60.2%)
Yes	68 (40.5%)	6 (33.3%)	74 (39.8%)
COPD			
No	158 (94.0%)	18 (100%)	176 (94.6%)
Yes	10 (6.0%)	0 (0%)	10 (5.4%)
Cerebrovascular accident			
No	164 (97.6%)	18 (100%)	182 (97.8%)
Yes	4 (2.4%)	0 (0%)	4 (2.2%)
Chronic heart disease			
No	159 (94.6%)	16 (88.9%)	175 (94.1%)
Yes	9 (5.4%)	2 (11.1%)	11 (5.9%)
Coronary heart disease			
No	163 (97.0%)	18 (100%)	181 (97.3%)
Yes	5 (3.0%)	0 (0%)	5 (2.7%)
Oncologic disease			
No	165 (98.2%)	18 (100%)	183 (98.4%)
Yes	3 (1.8%)	0 (0%)	3 (1.6%)
Metastatic cancer			
No	167 (99.4%)	18 (100%)	185 (99.5%)
Yes	1 (0.6%)	0 (0%)	1 (0.5%)
Chemotherapy			
No	168 (100%)	18 (100%)	186 (100%)
Yes	0 (0%)	0 (0%)	0 (0%)
Chronic kidney disease			
No	167 (99.4%)	17 (94.4%)	184 (98.9%)
Yes	1 (0.6%)	1 (5.6%)	2 (1.1%)
Hemodialysis			
No	166 (98.8%)	17 (94.4%)	183 (98.4%)
Yes	2 (1.2%)	1 (5.6%)	3 (1.6%)
Rheumatologic diseases			
No	168 (100%)	18 (100%)	186 (100%)
Yes	0 (0%)	0 (0%)	0 (0%)
HIV/AIDS			
No	163 (97.0%)	18 (100%)	181 (97.3%)
Yes	5 (3.0%)	0 (0%)	5 (2.7%)
Dementia			
No	167 (99.4%)	18 (100%)	185 (99.5%)
Yes	1 (0.6%)	0 (0%)	1 (0.5%)
Obesity			
No	137 (81.5%)	16 (88.9%)	153 (82.3%)
Yes	31 (18.5%)	2 (11.1%)	33 (17.7%)
Current tobacco use			
No	161 (95.8%)	18 (100%)	179 (96.2%)
Yes	7 (4.2%)	0 (0%)	7 (3.8%)
Former smoker			
No	158 (94.0%)	18 (100%)	176 (94.6%)
Yes	10 (6.0%)	0 (0%)	10 (5.4%)
Active tuberculosis			
No	165 (98.2%)	17 (94.4%)	182 (97.8%)
Yes	3 (1.8%)	1 (5.6%)	4 (2.2%)
Treated tuberculosis			
No	168 (100%)	18 (100%)	186 (100%)
Yes	0 (0%)	0 (0%)	0 (0%)
Cirrhosis			
No	167 (99.4%)	18 (100%)	185 (99.5%)
Yes	1 (0.6%)	0 (0%)	1 (0.5%)
Transplantation			
No	168 (100%)	18 (100%)	186 (100%)
Yes	0 (0%)	0 (0%)	0 (0%)
Aspirin or antiplatelet			
No	164 (97.6%)	17 (94.4%)	181 (97.3%)
Yes	4 (2.4%)	1 (5.6%)	5 (2.7%)
Anticoagulant			
No	166 (98.8%)	18 (100%)	184 (98.9%)
Yes	2 (1.2%)	0 (0%)	2 (1.1%)
SOFA			
Mean (SD)	4.26 (3.78)	6.94 (3.51)	4.52 (3.83)
Median [Min, Max]	3.00 [0, 13.0]	8.00 [1.00, 12.0]	3.00 [0, 13.0]
SAPS 3			
Mean (SD)	48.8 (18.2)	60.2 (14.6)	49.9 (18.1)
Median [Min, Max]	44.0 [18.0, 88.0]	59.5 [40.0, 82.0]	46.0 [18.0, 88.0]
CCI			
Mean (SD)	2.96 (2.04)	3.94 (2.29)	3.06 (2.08)
Median [Min, Max]	3.00 [0, 10.0]	4.00 [1.00, 8.00]	3.00 [0, 10.0]
WELLS			
Mean (SD)	0.0774 (0.289)	3.00 (0.767)	0.360 (0.938)
Median [Min, Max]	0 [0, 2.00]	3.00 [2.00, 4.00]	0 [0, 4.00]
SIC			
Mean (SD)	1.73 (0.887)	2.56 (0.922)	1.81 (0.921)
Median [Min, Max]	2.00 [0, 4.00]	2.00 [1.00, 4.00]	2.00 [0, 4.00]

CCI, Charlson Comorbidity Index; CT, computed tomography; Max, maximum; Min, minimum; SAPS 3, Simplified Acute Physiology Score 3; SD, standard deviation; SIC, sepsis‐induced coagulopathy; SOFA, sequential organ failure assessment.

The COVID‐19 severity was generally higher in the presence of DVT. The CCI and SIC scores showed minimal increases among the participants with DVT compared to the non‐DVT group. Additionally, the Wells score was higher in the presence of DVT. (Table 1).

None of the patients had a normal chest radiograph at admission, and only 1 had a normal CT scan. The most frequent tomographic images were consolidation, ground‐glass opacity, and pleural effusion, and there were no evident differences among the DVT groups in these findings (Table S1).

D‐dimer, procalcitonin, interleukin‐6, and C‐reactive protein levels were generally elevated in all participants. The D‐dimer, INR, and prothrombin level were the biomarkers that were markedly different in the DVT group (Table 2).

**Table 2:** Laboratory Baseline Data by Deep Venous Thrombosis Diagnosis

	Deep Venous Thrombosis		
	No (n = 168)	Yes (n = 18)	Overall (n = 186)
D‐dimer (ng/mL)			
Mean (SD)	2330 (3110)	7860 (4260)	2860 (3620)
Median [Min, Max]	1200 [30.0, 20000]	8400 [1600, 20000]	1300 [30.0, 20000]
INR			
Mean (SD)	1.09 (0.161)	1.21 (0.214)	1.10 (0.170)
Median [Min, Max]	1.04 [0.840, 1.95]	1.18 [0.900, 1.70]	1.05 [0.840, 1.95]
Prothrombin Time (sec)			
Mean (SD)	1.17 (1.02)	1.20 (0.216)	1.17 (0.976)
Median [Min, Max]	1.04 [0.860, 13.6]	1.17 [0.910, 1.78]	1.05 [0.860, 13.6]
Missing	15 (8.9%)	2 (11.1%)	17 (9.1%)
PTT (sec)			
Mean (SD)	27.7 (5.86)	29.4 (6.60)	27.8 (5.94)
Median [Min, Max]	27.0 [1.10, 50.9]	27.9 [19.4, 43.0]	27.0 [1.10, 50.9]
Missing	29 (17.3%)	3 (16.7%)	32 (17.2%)
Procalcitonin (ng/dL)			
Mean (SD)	2.28 (9.99)	3.00 (9.52)	2.35 (9.92)
Median [Min, Max]	0.175 [0.0300, 86.7]	0.340 [0.100, 40.8]	0.195 [0.0300, 86.7]
CRP (mg/L)			
Mean (SD)	13.4 (7.71)	12.2 (6.78)	13.3 (7.62)
Median [Min, Max]	12.9 [0.120, 40.1]	11.6 [1.97, 27.4]	12.6 [0.120, 40.1]
Erythrocyte sedimentation rate			
Mean (SD)	69.8 (39.1)	95.6 (32.9)	72.0 (39.2)
Median [Min, Max]	70.0 [0, 160]	91.0 [45.0, 160]	70.0 [0, 160]
Missing	22 (13.1%)	4 (22.2%)	26 (14.0%)
WBC count (10^3^/mm^3^)			
Mean (SD)	10900 (5360)	13900 (7830)	11200 (5690)
Median [Min, Max]	9910 [2700, 34200]	13000 [3130, 28600]	10100 [2700, 34200]
Platelets (10^3^/μL)			
Mean (SD)	273000 (124000)	267000 (105000)	273000 (122000)
Median [Min, Max]	252000 [47000, 756000]	274000 [96000, 474000]	257000 [47000, 756000]
Lymphocytes (10^3^/mm^3^)			
Mean (SD)	1350 (1210)	1270 (617)	1340 (1160)
Median [Min, Max]	1050 [15.0, 9580]	1260 [309, 2860]	1070 [15.0, 9580]
Missing	1 (0.6%)	0 (0%)	1 (0.5%)
Creatinine (mg/dL)			
Mean (SD)	1.39 (1.37)	2.13 (2.04)	1.46 (1.46)
Median [Min, Max]	1.04 [0.430, 11.1]	1.21 [0.500, 8.36]	1.04 [0.430, 11.1]
Amylase (U/L)			
Mean (SD)	63.9 (49.2)	74.4 (83.0)	64.9 (53.0)
Median [Min, Max]	47.0 [11.0, 341]	52.0 [26.0, 371]	47.0 [11.0, 371]
Missing	12 (7.1%)	2 (11.1%)	14 (7.5%)
Lipase (U/L)			
Mean (SD)	45.5 (45.6)	60.5 (72.7)	46.9 (48.7)
Median [Min, Max]	34.5 [7.00, 458]	26.0 [10.0, 266]	33.0 [7.00, 458]
Missing	10 (6.0%)	2 (11.1%)	12 (6.5%)
ALT (U/L)			
Mean (SD)	83.5 (265)	64.7 (68.4)	81.6 (252)
Median [Min, Max]	37.5 [5.00, 2820]	33.5 [10.0, 226]	37.0 [5.00, 2820]
Missing	8 (4.8%)	0 (0%)	8 (4.3%)
AST (U/L)			
Mean (SD)	64.1 (115)	72.9 (43.9)	65.0 (110)
Median [Min, Max]	39.0 [8.00, 1140]	63.5 [29.0, 185]	40.5 [8.00, 1140]
Missing	10 (6.0%)	0 (0%)	10 (5.4%)
Bilirubin (mg/dL)			
Mean (SD)	0.765 (2.25)	0.674 (0.513)	0.757 (2.14)
Median [Min, Max]	0.440 [0.140, 28.6]	0.440 [0.190, 1.81]	0.440 [0.140, 28.6]
Missing	4 (2.4%)	1 (5.6%)	5 (2.7%)
IL‐6 (pg/mL)			
Mean (SD)	56.7 (73.1)	69.5 (82.9)	57.9 (73.5)
Median [Min, Max]	27.5 [0.100, 352]	47.8 [1.50, 226]	29.1 [0.100, 352]
Missing	110 (65.5%)	12 (66.7%)	122 (65.6%)
Ferritin (ng/mL)			
Mean (SD)	836 (496)	767 (585)	827 (505)
Median [Min, Max]	810 [50.3, 1500]	676 [37.2, 1500]	810 [37.2, 1500]
Missing	103 (61.3%)	8 (44.4%)	111 (59.7%)

ALT, alanine aminotransferase; AST, aspartate aminotransferase; CRP, C‐reactive protein; IL‐6, interleukin‐6; INR, international normalized ratio; Max, maximum; Min, minimum; SD, standard deviation; PTT, partial thromboplastin time activated; WBC, white blood cell.

The Wells Score ranged from zero to 4 among the participants. The score was sensitive, so there were no participants with DVT with a score of 0 or 1, and specific, as there were no participants without DVT with scores of 3 or 4. The DVT score with the highest pair of sensitivity and specificity was 2. When considering a score of 2 or higher to identify the occurrence of DVT, the sensitivity and specificity confidence intervals were observed to be greater than 80%. (Table 3) Additional scores were tested to predict DVT, such as the SIC score (Table S2), SAPS 3 score (Table S3), CCI (Table S4), and SOFA score (Table S5); however, they all performed poorly to moderately.

**Table 3:** The Diagnostic Accuracy of Wells Score Performed at Admission for Detecting DVT Among COVID‐19 Patients

Wells Score	D	ND	TP	FN	FP	TN	Sensitivity	Se.inf.cl	Se.sup.cl	Specificity	Sp.inf.cl	Sp.sup.cl
0	0	156	18	0	168	1	**1.000**	0.824	1.000	**0.006**	0.001	0.033
1	0	11	18	0	12	156	**1.000**	0.824	1.000	**0.929**	0.879	0.959
2	5	1	18	0	1	167	**1.000**	0.824	1.000	**0.994**	0.967	0.999
3	8	0	13	5	0	168	**0.722**	0.491	0.875	**1.000**	0.978	1.000
4	5	0	5	13	0	168	**0.278**	0.125	0.509	**1.000**	0.978	1.000

Cl, 95% confidence limit; D, with deep vein thrombosis; FN, false negative; FP, false positive; Inf, inferior; ND, without deep vein thrombosis; Se, sensitivity; Sp, specificity; TSup, superior; N, true negative; TP, true positive.

Hospital mortality and the need for mechanical ventilation were significantly higher among participants with DVT. In contrast, the use of vasopressors was slightly higher in the DVT group, while the length of hospital stay and hemodialysis outcomes were similar among the DVT groups (Table 4). Additionally, regression models taking the outcome as the length of stay (Table S6), hemodialysis (Table S7), or length of mechanical ventilation (Table S8) as outcomes were not significant, either with DVT as a univariate predictor or with DVT adjusted for the SAPS 3 score.

**Table 4:** Intra‐Hospital Outcomes by Deep Venous Thrombosis Diagnosis

	Deep Venous Thrombosis		
	No (n = 168)	Yes (n = 18)	Overall (n = 186)
Length of stay (days)			
Mean (SD)	16.6 (13.8)	19.8 (16.4)	16.9 (14.0)
Median [Min, Max]	13.0 [2.00, 87.0]	15.0 [3.00, 73.0]	13.0 [2.00, 87.0]
Death			
No	111 (66.1%)	4 (22.2%)	115 (61.8%)
Yes	57 (33.9%)	14 (77.8%)	71 (38.2%)
Ventilatory support			
No support	17 (10.1%)	1 (5.6%)	18 (9.7%)
Inhalatory O_2_	72 (42.9%)	6 (33.3%)	78 (41.9%)
Facial mask O_2_	47 (28.0%)	3 (16.7%)	50 (26.9%)
NIV	4 (2.4%)	1 (5.6%)	5 (2.7%)
Mechanical support	28 (16.7%)	7 (38.9%)	35 (18.8%)
Nasal catheter (L/min)			
Mean (SD)	5.89 (11.7)	4.75 (3.10)	5.81 (11.3)
Median [Min, Max]	4.50 [2.00, 89.0]	4.00 [2.00, 9.00]	4.50 [2.00, 89.0]
Missing	114 (67.9%)	14 (77.8%)	128 (68.8%)
Oxygen mask (L/min)			
Mean (SD)	9.39 (3.94)	NA (NA)	9.39 (3.94)
Median [Min, Max]	10.0 [3.00, 15.0]	NA [NA, NA]	10.0 [3.00, 15.0]
Missing	130 (77.4%)	18 (100%)	148 (79.6%)
Norepinephrine			
No	141 (83.9%)	12 (66.7%)	153 (82.3%)
Yes	27 (16.1%)	6 (33.3%)	33 (17.7%)
Vasopressin			
No	157 (93.5%)	16 (88.9%)	173 (93.0%)
Yes	11 (6.5%)	2 (11.1%)	13 (7.0%)
Dobutamine			
No	162 (96.4%)	18 (100%)	180 (96.8%)
Yes	6 (3.6%)	0 (0%)	6 (3.2%)
Nitroglycerin			
No	168 (100%)	18 (100%)	186 (100%)
Yes	0 (0%)	0 (0%)	0 (0%)
Nitroprusside			
No	160 (95.2%)	18 (100%)	178 (95.7%)
Yes	8 (4.8%)	0 (0%)	8 (4.3%)
Hemodialysis			
No	148 (88.1%)	14 (77.8%)	162 (87.1%)
Yes	20 (11.9%)	4 (22.2%)	24 (12.9%)

Max, maximum; Min, minimum; NIV, noninvasive ventilation; SD, standard deviation.

DVT was a relevant predictor of both the need for mechanical ventilation and hospital mortality (Table 5). In both analyses, the SAPS 3 severity score was a stronger predictor than DVT. Nevertheless, the presence of DVT explained a relevant amount of variation in the survival models and revealed that participants with DVT had twice the hazards compared to participants without DVT, even after adjusting for the SAPS 3 severity score (Table 5).

**Table 5:** Cox Proportional Hazards Models' Coefficients and Hazard Ratios of DVT for Different Outcomes Adjusted for SAPS 3 Among COVID‐19 Patients

Outcome	Variables	Effect	SE	Lower 0.95	Upper 0.95
Mechanical ventilation^a^, ^b^	SAPS 3	1.494	0.227	1.050	1.938
	Hazard ratio	4.455		2.857	6.945
	Deep vein thrombosis—Yes:No	0.875	0.396	0.099	1.651
	Hazard ratio	2.399		1.104	5.214
Death^c^	SAPS 3	1.530	0.243	1.053	2.007
	Hazard ratio	4.619		2.867	7.441
	Deep vein thrombosis—Yes:No	1.073	0.300	0.484	1.661
	Hazard ratio	2.923		1.622	5.266

Lower, lower confidence limit; Upper, upper confidence limit; SE, standard error.

^a^ R^2^, 0.270.

^b^ Excluding participants requiring mechanical ventilation at admission.

^c^ R^2^, 0.262.

**Figure 1:**
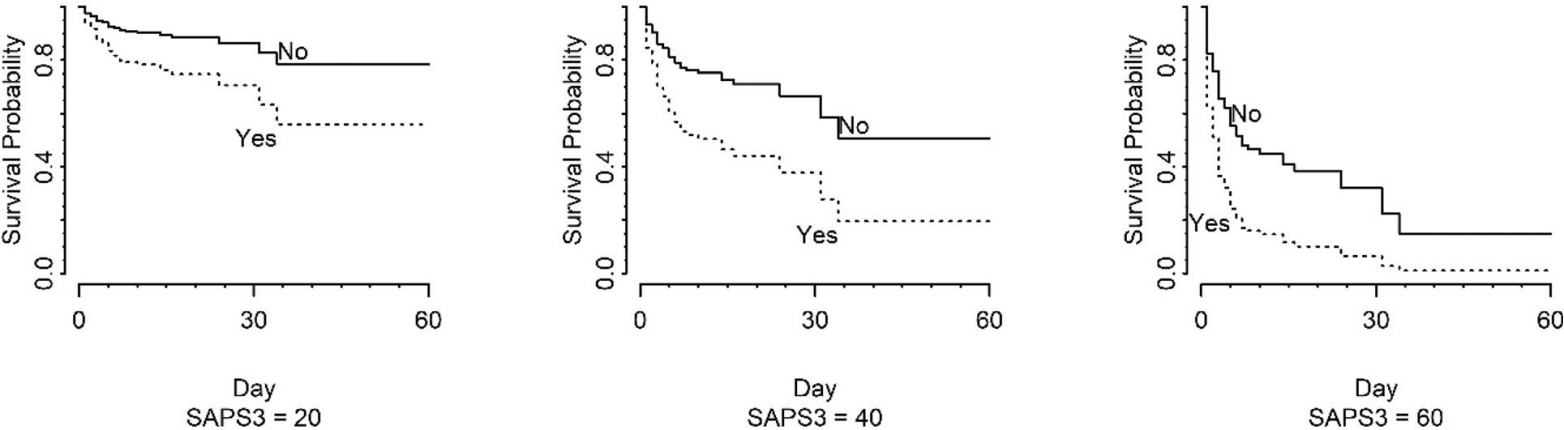
Cox regression survival curves of patients with and without deep vein thrombosis adjusted for SAPS 3 score, taking mechanical ventilation onset as outcome (excluding participants on mechanical ventilation on admission).

At a SAPS 3 score as low as 20, participants with DVT had a survival probability of approximately 55% for remaining free of mechanical ventilation for up to 30 days. Additionally, as the SAPS 3 score increased to 40 and 60, the survival probability decreased to approximately 28 and 5%, respectively, on day 30 for the same participants (Figure 1).

**Figure 2:**
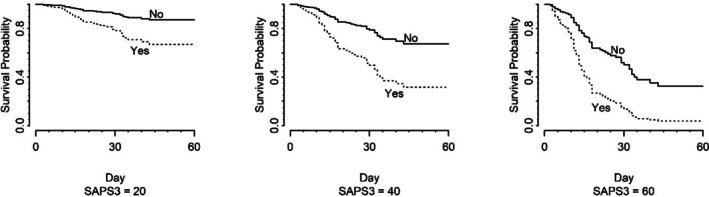
Cox regression survival curves of patients with and without deep vein thrombosis, adjusted for SAPS 3 score, taking death as the outcome.

At a SAPS 3 score as low as 20, the survival probability (death outcome) up to 30 days was approximately 80% for the participants with DVT. Additionally, as the SAPS 3 score increased to 40 and 60, the survival probability decreased to approximately 58 and 20%, respectively, at day 30 for the same participants (Figure 2).

In addition, we provide a web calculator available at https://pedrobrasil.shinyapps.io/INDWELL/. This web calculator is part of the results, showing findings that cannot be presented as a static manuscript. It was developed from the Wells performance results and from the survival models. One can visit the web calculator and enter the data from their patient, and the web calculator returns the Wells score and its predictive value. In the prognosis calculator, one may enter the SPAS 3 score and the DVT diagnosis; then the calculator returns the adjusted survival (overall death) and the survival probability taking mechanical ventilation as the outcome.

## Discussion

The main results to be discussed are as follows: 1) the cumulative incidence of DVT and its respective 95% confidence interval was 0.097 [0.062–0.148]. 2) The DVT diagnosis by Wells' score proved to be accurate for COVID‐19 patients. 3) DVT proved to be a prognostic marker in COVID‐19 patients, even when adjusted by the SAPS 3 severity score.

A few studies have addressed the incidence of VTE in Coronavirus Disease‐2019 (COVID‐19) as a phenomenon that still lacks clarity.^18^ Conversely, other researchers assert that there exists a widely accepted consensus regarding the elevated risk of thromboembolic events, such as DVT, associated with SARS‐CoV‐2 infection.^62^ Nevertheless, studies estimated the incidence of DVT in critically ill patients with COVID‐19 from 9.46 to 46%.^2^, ^7^, ^8^, ^9^ The DVT risk could be as high as 85% depending on the setting. This large variability may be related to the differences in patient sampling, hospital settings, and diagnostic protocols for VTE. Additionally, the risk of DVT appears to be higher in ICUs despite prophylaxis protocols.^18^

A systematic review of predictive scores for the diagnosis of PTE in COVID‐19 patients published in February 2022 focused on the need to establish new prediction rules, specifically developed and validated for estimating the risk of pulmonary embolism, drawing attention to the limited number of studies on this subject.^63^ Although certain studies have highlighted that the incidence of DVT is decreasing with progressive knowledge regarding COVID‐19 care, the Wells score has not yet been validated for the population with COVID‐19.^64^ However, since then, there is evidence showing it is possible to screen and perform DVT diagnostic investigation in COVID‐19 patients using a combination of the Wells score and D‐dimer.^40^

There is a difference in the prevalence of DVT or PTE in the hospital setting, from studies including wards, wards plus ICUs, and with or without screening at admission.^65^ It is likely that with these studies with different approaches, diagnostic scores would perform differently. There are some studies investigating the scores' performance for PTE (such as Geneva and Wells scores) showing that these have poor performances,^66^ but combining the clinical scores with other biomarkers may increase overall diagnostic performance.^64^, ^66^, ^67^ However, for DVT diagnosis, we were able to find 2 similar studies to ours, and the main differences are that this study approach had data from chart reviews, and US screening was not performed systematically at admission. Their findings show a very poor balance between sensitivity and specificity, even when combining the Wells score with other biomarkers, such as D‐dimer. From this evidence, one should either choose high sensitivity with a low cut‐off (such as 2), or choose high specificity with a high cut‐off (such as 4 or higher).^40^, ^68^ An important aspect that strengthens the importance of Wells score validation within our study is the evidence that it is possible to accurately predict DVT diagnosis before the US is performed, and the evidence of DVT as a marker of COVID‐19 poor prognosis.

There is an association between COVID‐19 and VTE. Certain researchers suggest that a cytokine storm is a key factor in the fast deterioration of COVID‐19, proposing that the knowledge of the time and procedure for blocking the cytokine storm and the time for initiation of anti‐inflammatory therapy is critical for reducing the death rate due to COVID‐19.^69^, ^70^ The concept of cytokine storm has gradually emerged because of the extremely high mortality associated with multi‐organ failure described in the literature, and it seems it is not exclusive to COVID‐19.

Laboratory experiments conducted on avian Influenza A (H5N1) viruses suggest that virus‐induced cytokine dysregulation contributes to disease severity.^71^ There is a report on acute lung injury and acute respiratory distress syndrome caused partially by host immune responses, suggesting that corticosteroids suppress lung inflammation, which is the case with SARS‐CoV infection, as well as influenza, for which systemic inflammation is associated with adverse outcomes.^72^

The laboratory tests related to clot dysfunction in our study were more frequently abnormal in patients with DVT. Hypercoagulability is a hallmark of inflammation. Pro‐inflammatory cytokines, such as interleukin (IL)‐6, IL‐17A, and tumor necrosis factor, are frequently elevated in patients with severe outcomes. Therefore, inflammatory cytokines are critically involved in abnormal clot formation and platelet hyperactivation and play a crucial role in the downregulation of important physiological anticoagulant mechanisms. This suggests that anticoagulant drugs started immediately after confirming SARS‐CoV‐2 infection to prevent the occurrence of life‐threatening complications may be beneficial.^62^

Mainly in the beginning of the COVID‐19 pandemic, there were some differences in guidelines regarding DVT prevention and treatment in COVID‐19 patients.^46^, ^73^, ^74^, ^75^, ^76^ The current consensus in the medical literature is that the treatment of DVT in critically ill patients with COVID‐19 should follow the same principles as for DVT in other critically ill populations. In general, the recommended approach is therapeutic anticoagulation with parenteral agents (Low Molecular Weight Heparin or Unfractionated Heparin), with careful attention to individual patient factors and standard duration of therapy.^77^ Routine monitoring of coagulation parameters (eg, anti‐Xa levels for LMWH, aPTT for UFH) is advised in the ICU, especially in patients with fluctuating renal function, obesity, or other factors affecting drug metabolism.^78^

One of the limitations of this research is the referral involved in the INI/FIOCRUZ hospitalizations. The referral may have previously selected patients with more severe conditions to be transferred to INI/FIOCRUZ, as these patients would be more in need of specialized care than others waiting for hospitalization, despite the observed mortality being similar to the mortality reported elsewhere. Nevertheless, if this were the case, then DVT incidence might be overestimated. On the other hand, as no vaccines were available at the time and mortality rates were high, if transfers were not timely to provide appropriate care, it is possible that severe cases died before they could be observed in the study. If this were the case, DVT incidence might be underestimated. Nevertheless, the setting was very representative of real‐life situations at the time, and this may be an issue when making inferences about DVT incidence in diverse settings.

An additional limitation is that COVID‐19 variants of concern changed very rapidly. While the study was going on, the Wild, Zeta, and Alpha variants circulated. Later, other variants were preferentially observed. It means that the clinical manifestations and progression to critical illness currently may have other frequencies, and the DVT incidence may require an update in order to better infer the current epidemiological setting.

Finally, the limitation of the sample size made it hard to detect potential relationships of DVT with some secondary outcomes, such as hemodialysis requirement and time using ventilation support. Additionally, a study carried out in a single center may make it difficult to generalize the results, since different ICUs, where there is a potential for applicability, may not share the same setting characteristics as our research setting.

## Conclusions

The incidence of DVT in this study was not as high as that reported elsewhere. The evidence suggests that it is possible to accurately diagnose DVT using the Wells score in critically ill patients with COVID‐19, regardless of the D‐dimer test. The Wells score performance was highly accurate and has the potential to replace US in the ICU admission screening, specifically in settings where US is unavailable. DVT increases the severity in critically ill patients with COVID‐19 infection, increasing the risk of the need for mechanical ventilation, as well as the risk of mortality. Therefore, there is evidence to provide a rationale for guiding anticoagulant prophylaxis and therapy.

## Supporting information


**Data S1.** Supporting information.
**Table S1.** Lung images baseline data by deep venous thrombosis diagnosis.
**Table S2.** SIC score diagnostic performance in DVT among COVID‐19 patients.
**Table S3.** SAPS 3 score diagnostic performance in DVT among COVID‐19 patients.
**Table S4.** Charlson comorbidity index diagnostic performance in DVT among COVID‐19 patients.
**Table S5.** SOFA score diagnostic performance in DVT among COVID‐19 patients.
**Table S6.** Least square linear model DVT and SAPS 3 coefficients predicting log length of stay among Covid‐19 patients.
**Table S7.** Cox proportional hazards model coefficients in hazard ratios of DVT for hemodialysis adjusted for SAPS 3 among Covid‐19 patients.
**Table S8.** Least square linear model DVT and SAPS 3 coefficients predicting log length of mechanical ventilation among Covid‐19 patients.

## Data Availability

The dataset supporting the conclusions of this article will be included at the institutional repository at https://www.arca.fiocruz.br/?locale‐attribute=en after manuscript publication.

## References

[1] Ministério da Saúde . Boletim Epidemiológico N^o^ 133—Boletim COE Coronavírus—Português (Brasil) 2022. Accessed October 12, 2022. https://www.gov.br/saude/pt‐br/centrais‐de‐conteudo/publicacoes/boletins/epidemiologicos/covid‐19/2022/boletim‐epidemiologico‐no‐133‐boletim‐coe‐coronavirus/view.

[2] Santoliquido A , Porfidia A , Nesci A , et al. Incidence of deep vein thrombosis among non‐ICU patients hospitalized for COVID‐19 despite pharmacological thromboprophylaxis. J Thromb Haemost 2020; 18:2358–2363. 10.1111/jth.14992. 32633068 PMC7361278

[3] Li J , Huang DQ , Zou B , et al. Epidemiology of COVID‐19: a systematic review and meta‐analysis of clinical characteristics, risk factors, and outcomes. J Med Virol 2021; 93:1449–1458. 10.1002/jmv.26424. 32790106 PMC7436673

[4] Berlin DA , Gulick RM , Martinez FJ . Severe Covid‐19. N Engl J Med 2020; 383:2451–2460. 10.1056/NEJMcp2009575. 32412710

[5] Li B , Yang J , Zhao F , et al. Prevalence and impact of cardiovascular metabolic diseases on COVID‐19 in China. Clin Res Cardiol 2020; 109:531–538. 10.1007/s00392-020-01626-9. 32161990 PMC7087935

[6] Jasinowodolinski D , Marins Filisbino M , Guedes Baldi B . COVID‐19 pneumonia: a risk factor for pulmonary thromboembolism? J Bras Pneumol 2020; 46:e20200168. 10.36416/1806-3756/e20200168. 32490908 PMC7567618

[7] Franco‐Moreno A , Herrera‐Morueco M , Mestre‐Gómez B , et al. Incidence of deep venous thrombosis in patients with COVID‐19 and pulmonary embolism. J Ultrasound Med 2021; 40:1411–1416. 10.1002/jum.15524. 33017480 PMC7675470

[8] Baccellieri D , Bertoglio L , Apruzzi L , et al. Incidence of deep venous thrombosis in COVID‐19 hospitalized patients during the first peak of the Italian outbreak. Phlebology 2021; 36:375–383. 10.1177/0268355520975592. 33241746

[9] Pieralli F , Pomero F , Giampieri M , et al. Incidence of deep vein thrombosis through an ultrasound surveillance protocol in patients with COVID‐19 pneumonia in non‐ICU setting: a multicenter prospective study. PLoS One 2021; 16:e0251966. 10.1371/journal.pone.0251966. 34015018 PMC8136742

[10] Cunha MJS , Pinto CAV , Guerra JC d C , et al. Incidence, diagnosis, treatment methods, and outcomes of clinically suspected venous thromboembolic disease in patients with COVID‐19 in a quaternary hospital in Brazil. J Vasc Bras 2021; 20:e20200203. 10.1590/1677-5449.200203. 34188671 PMC8210641

[11] Pereira de Godoy JM , Russeff GJ d S , Cunha CH , et al. Increased prevalence of deep vein thrombosis and mortality in patients with Covid‐19 at a referral center in Brazil. Phlebology 2022; 37:21–25. 10.1177/02683555211041931. 34494482 PMC8829736

[12] Pellegrini JAS , Rech TH , Schwarz P , et al. Incidence of venous thromboembolism among patients with severe COVID‐19 requiring mechanical ventilation compared to other causes of respiratory failure: a prospective cohort study. J Thromb Thrombolysis 2021; 52:482–492. 10.1007/s11239-021-02395-6. 33599858 PMC7890785

[13] Pereira De Godoy JM , Da Silva Russeff GJ , Hungaro Cunha C , et al. Mortality and change in the prevalence of deep vein thrombosis associated with SARS‐CoV‐2 P.1 variant. Cureus 2022; 14:e26668. 10.7759/cureus.26668. 35949793 PMC9357448

[14] Mai V , Tan BK , Mainbourg S , et al. Venous thromboembolism in COVID‐19 compared to non‐COVID‐19 cohorts: a systematic review with meta‐analysis. Vascul Pharmacol 2021; 139:106882. 10.1016/j.vph.2021.106882. 34087481 PMC8169236

[15] Tufano A , Rendina D , Abate V , et al. Venous thromboembolism in COVID‐19 compared to non‐COVID‐19 cohorts: a systematic review with meta‐analysis. J Clin Med 2021; 10:4925. 10.3390/jcm10214925. 34768445 PMC8584903

[16] Longchamp G , Manzocchi‐Besson S , Longchamp A , et al. Proximal deep vein thrombosis and pulmonary embolism in COVID‐19 patients: a systematic review and meta‐analysis. Thromb J 2021; 19:15. 10.1186/s12959-021-00266-x. 33750409 PMC7942819

[17] Tan BK , Mainbourg S , Friggeri A , et al. Arterial and venous thromboembolism in COVID‐19: a study‐level meta‐analysis. Thorax 2021; 76:970–979. 10.1136/thoraxjnl-2020-215383. 33622981

[18] Porfidia A , Valeriani E , Pola R , et al. Venous thromboembolism in patients with COVID‐19: systematic review and meta‐analysis. Thromb Res 2020; 196:67–74. 10.1016/j.thromres.2020.08.020. 32853978 PMC7420982

[19] Voicu S , Ketfi C , Stépanian A , et al. Pathophysiological processes underlying the high prevalence of deep vein thrombosis in critically ill COVID‐19 patients. Front Physiol 2021; 11:608788. 10.3389/fphys.2020.608788. 33488398 PMC7820906

[20] Lobbes H , Mainbourg S , Mai V , et al. Risk factors for venous thromboembolism in severe COVID‐19: a study‐level meta‐analysis of 21 studies. Int J Environ Res Public Health 2021; 18:12944. 10.3390/ijerph182412944. 34948552 PMC8700787

[21] Shah A , Donovan K , McHugh A , et al. Thrombotic and haemorrhagic complications in critically ill patients with COVID‐19: a multicentre observational study. Crit Care 2020; 24:561. 10.1186/s13054-020-03260-3. 32948243 PMC7499016

[22] Koleilat I , Galen B , Choinski K , et al. Clinical characteristics of acute lower extremity deep venous thrombosis diagnosed by duplex in patients hospitalized for coronavirus disease 2019. J Vasc Surg Venous Lymphat Disord 2021; 9:36–46. 10.1016/j.jvsv.2020.06.012. 32593770 PMC7315975

[23] Raskob GE , Spyropoulos AC , Cohen AT , et al. Association between asymptomatic proximal deep vein thrombosis and mortality in acutely ill medical patients. J Am Heart Assoc 2021; 10:e019459. 10.1161/JAHA.120.019459. 33586478 PMC8174250

[24] de Godoy JMP , da Silva MOM , Santos HA , et al. Mortality, deep vein thrombosis, and D‐dimer levels in patients with COVID‐19. Cor Vasa 2022; 64:399–402. 10.33678/cor.2022.018.

[25] Hill JB , Garcia D , Crowther M , et al. Frequency of venous thromboembolism in 6513 patients with COVID‐19: a retrospective study. Blood Adv 2020; 4:5373–5377. 10.1182/bloodadvances.2020003083. 33137202 PMC7656921

[26] Chiesa AF , Previsdomini M , Valenti E , et al. Prevalence and risk factors for venous thromboembolic events in critically ill patients with SARS‐CoV‐2 infection: a prospective observational study. Minerva Anestesiol 2021; 87:1330–1337. 10.23736/S0375-9393.21.15510-5. 34633166

[27] Barnett N , Leith D , Govind D , et al. Prevalence of pulmonary embolism and deep venous thrombosis during the COVID‐19 pandemic in an intensive care unit cohort: a service evaluation. Br J Anaesth 2022; 129:e124–e126. 10.1016/j.bja.2022.07.040. 36055821 PMC9365863

[28] Hunter M , Lurbet MF , Parodi J , et al. Deep venous thrombosis incidence in patients with COVID‐19 acute respiratory distress syndrome, under intermediate dose of chemical thromboprophylaxis. Medicina (B Aires) 2022; 82:181–184. 35417380

[29] Trigonis RA , Holt DB , Yuan R , et al. Incidence of venous thromboembolism in critically ill coronavirus disease 2019 patients receiving prophylactic anticoagulation. Crit Care Med 2020; 48:e805–e808. 10.1097/CCM.0000000000004472. 32618699 PMC7314344

[30] De Pereira Godoy JM , Russeff GJDS , Costa CH , et al. Mortality of patients infected by COVID‐19 with and without deep‐vein thrombosis. Medicines (Basel) 2021; 8:75. 10.3390/medicines8120075. 34940287 PMC8708913

[31] Correia RM , Santos BC , Carvalho AAG , et al. Vascular complications in 305 severely ill patients with COVID‐19: a cohort study. Sao Paulo Med J 2023; 141:e2022171. 10.1590/1516-3180.2022.0171.r2.17102022. PMC10065094 36541953

[32] Gil‐Sala D , Riera C , García‐Reyes M , et al. Mortality and bleeding complications of COVID‐19 critically ill patients with venous thromboembolism. Int Angiol 2022; 41:1–8. 10.23736/S0392-9590.21.04704-0. 34751541

[33] Wells PS , Anderson DR , Bormanis J , et al. Value of assessment of pretest probability of deep‐vein thrombosis in clinical management. Lancet 1997; 350:1795–1798. 10.1016/S0140-6736(97)08140-3. 9428249

[34] Albricker ACL , Freire CMV , dos Santos SN , et al. Diretriz Conjunta sobre Tromboembolismo Venoso – 2022. Arq Bras Cardiol 2022; 118:797–857. 10.36660/abc.20220213. 35508060 PMC9007000

[35] Bates SM , Jaeschke R , Stevens SM , et al. Diagnosis of DVT: antithrombotic therapy and prevention of thrombosis, 9th ed: American College of Chest Physicians Evidence‐Based Clinical Practice Guidelines. Chest 2012; 141:e351S–e418S. 10.1378/chest.11-2299. 22315267 PMC3278048

[36] Projeto Diretrizes SBACV – Sociedade Brasileira de Angiologia e Cirurgia Vascular . Trombose venosa profunda diagnóstico e tratamento. 2015.

[37] Silveira PC , Ip IK , Goldhaber SZ , et al. Performance of Wells score for deep vein thrombosis in the inpatient setting. JAMA Intern Med 2015; 175:1112–1117. 10.1001/jamainternmed.2015.1687. 25985219

[38] Trihan J‐E , Adam M , Jidal S , et al. Performance of the Wells score in predicting deep vein thrombosis in medical and surgical hospitalized patients with or without thromboprophylaxis: the R‐WITT study. Vasc Med 2021; 26:288–296. 10.1177/1358863X21994672. 33749393

[39] Chopard R , Albertsen IE , Piazza G . Diagnosis and treatment of lower extremity venous thromboembolism: a review. JAMA 2020; 324:1765–1776. 10.1001/jama.2020.17272. 33141212

[40] Raj K , Chandna S , Doukas SG , et al. Combined use of Wells scores and D‐dimer levels for the diagnosis of deep vein thrombosis and pulmonary embolism in COVID‐19: a retrospective cohort study. Cureus 2021; 13:e17687. 10.7759/cureus.17687. 34650862 PMC8487632

[41] Chen S , Zhang D , Zheng T , et al. DVT incidence and risk factors in critically ill patients with COVID‐19. J Thromb Thrombolysis 2021; 51:33–39. 10.1007/s11239-020-02181-w. 32607652 PMC7324310

[42] Longchamp A , Longchamp J , Manzocchi‐Besson S , et al. Venous thromboembolism in critically ill patients with COVID‐19: results of a screening study for deep vein thrombosis. Res Pract Thromb Haemost 2020; 4:842–847. 10.1002/rth2.12376. 32685893 PMC7272794

[43] Galien S , Hultström M , Lipcsey M , et al. Point of care ultrasound screening for deep vein thrombosis in critically ill COVID‐19 patients, an observational study. Thromb J 2021; 19:38. 10.1186/s12959-021-00272-z. 34078399 PMC8170442

[44] Eksioglu M , Azapoglu Kaymak B , Elhan AH , et al. A comparative analysis of the impact of severe acute respiratory syndrome coronavirus 2 infection on the performance of clinical decision‐making algorithms for pulmonary embolism. J Clin Med 2024; 13:7008. 10.3390/jcm13237008. 39685466 PMC11642087

[45] Özhan A , Bastopcu M . Factors associated with positive thrombus findings at ultrasonography in COVID‐19 ward patients who underwent imaging for suspected deep vein thrombosis under prophylactic anticoagulation. J Vasc Surg Venous Lymphat Disord 2022; 10:811–817. 10.1016/j.jvsv.2022.02.012. 35218956 PMC8864886

[46] Cuker A , Tseng EK , Nieuwlaat R , et al. American society of hematology living guidelines on the use of anticoagulation for thromboprophylaxis in patients with COVID‐19: May 2021 update on the use of intermediate‐intensity anticoagulation in critically ill patients. Blood Adv 2021; 5:3951–3959. 10.1182/bloodadvances.2021005493. 34474482 PMC8416320

[47] Ren B , Yan F , Deng Z , et al. Extremely high incidence of lower extremity deep venous thrombosis in 48 patients with severe COVID‐19 in Wuhan. Circulation 2020; 142:181–183. 32412320 10.1161/CIRCULATIONAHA.120.047407

[48] Perazzo H , Cardoso SW , Ribeiro MPD , et al. In‐hospital mortality and severe outcomes after hospital discharge due to COVID‐19: a prospective multicenter study from Brazil. Lancet Reg Health Am 2022; 11:100244. 10.1016/j.lana.2022.100244. 35434696 PMC9001143

[49] WHO . Emergency use ICD codes for COVID‐19 disease outbreak 2020. Accessed October 19, 2022. https://www.who.int/standards/classifications/classification‐of‐diseases/emergency‐use‐icd‐codes‐for‐covid‐19‐disease‐outbreak.

[50] Fiocruz . Leonel F. Brasil celebra um ano da vacina contra a Covid‐19. 2022. Accessed October 16, 2022. https://portal.fiocruz.br/noticia/brasil-celebra-um-ano-da-vacina-contra-covid-19.

[51] Wells P S , Hirsh J , Anderson D R , et al. Accuracy of clinical assessment of deep‐vein thrombosis. Lancet 1995; 345:1326–1330. 10.1016/S0140-6736(95)92535-X. 7752753

[52] Modi S , Deisler R , Gozel K , et al. Wells criteria for DVT is a reliable clinical tool to assess the risk of deep venous thrombosis in trauma patients. World J Emerg Surg 2016; 11:24. 10.1186/s13017-016-0078-1. 27279896 PMC4898382

[53] Charlson ME , Carrozzino D , Guidi J , et al. Charlson comorbidity index: a critical review of clinimetric properties. Psychother Psychosom 2022; 91:8–35. 10.1159/000521288. 34991091

[54] Raschke RA , Agarwal S , Rangan P , et al. Discriminant accuracy of the SOFA score for determining the probable mortality of patients with COVID‐19 pneumonia requiring mechanical ventilation. JAMA 2021; 325:1469–1470. 10.1001/jama.2021.1545. 33595630 PMC7890534

[55] Silva Junior JM , Malbouisson LMS , Nuevo HL , et al. Aplicabilidade do escore fisiológico agudo simplificado (SAPS 3) em hospitais brasileiros. Rev Bras Anestesiol 2010; 60:20–31. 10.1590/S0034-70942010000100003. 20169260

[56] Soares M , Silva UVA , Teles JMM , et al. Validation of four prognostic scores in patients with cancer admitted to Brazilian intensive care units: results from a prospective multicenter study. Intensive Care Med 2010; 36:1188–1195. 10.1007/s00134-010-1807-7. 20221751

[57] Nassar AP , Malbouisson LS , Moreno R . Evaluation of simplified acute physiology score 3 performance: a systematic review of external validation studies. Crit Care 2014; 18:R117. 10.1186/cc13911. 24906651 PMC4230997

[58] Pasinato VF , Franzosi OS , Loss SH , et al. SAPS 3 in the modified NUTrition RIsk in the critically ill score has comparable predictive accuracy to APACHE II as a severity marker. Rev Bras Ter Intensiva 2021; 33:394–400. 10.5935/0103-507X.20210064. 35107550 PMC8555402

[59] Tanaka C , Tagami T , Kudo S , et al. Validation of sepsis‐induced coagulopathy score in critically ill patients with septic shock: post hoc analysis of a nationwide multicenter observational study in Japan. Int J Hematol 2021; 114:164–171. 10.1007/s12185-021-03152-4. 33895968 PMC8067778

[60] Fischer EA , Kinnear B , Sall D , et al. Hospitalist‐operated compression ultrasonography: a point‐of‐care ultrasound study (HOCUS‐POCUS). J Gen Intern Med 2019; 34:2062–2067. 10.1007/s11606-019-05120-5. 31388904 PMC6816719

[61] Canon Medical Systems . Ultrasound System Transducer Viamo C100 2020. Accessed October 19, 2022. https://ch.medical.canon/wp‐content/uploads/sites/33/2020/05/Cleaning‐guidance‐Viamo‐c100.pdf.

[62] Tudoran C , Tudoran M , Abu‐Awwad A , et al. Spontaneous hematomas and deep vein thrombosis during the recovery from a SARS‐CoV‐2 infection: case report and literature review. Medicina (Kaunas) 2022; 58:230. 10.3390/medicina58020230. 35208553 PMC8878215

[63] Rindi LV , Al Moghazi S , Donno DR , et al. Predictive scores for the diagnosis of pulmonary embolism in COVID‐19: a systematic review. Int J Infect Dis 2022; 115:93–100. 10.1016/j.ijid.2021.11.038. 34848375 PMC8627287

[64] Cho ES , McClelland PH , Cheng O , et al. Utility of d‐dimer for diagnosis of deep vein thrombosis in coronavirus disease‐19 infection. J Vasc Surg Venous Lymphat Disord 2021; 9:47–53. 10.1016/j.jvsv.2020.07.009. 32738407 PMC7390766

[65] Silva B , Jorge C , Rigueira J , et al. Wells and Geneva decision rules to predict pulmonary embolism: can we use them in Covid‐19 patients? Eur Heart J 2021; 22:jeab111.009. 10.1093/ehjci/jeab111.009.

[66] Kirsch B , Aziz M , Kumar S , et al. Wells score to predict pulmonary embolism in patients with coronavirus disease 2019. Am J Med 2021; 134:688–690. 10.1016/j.amjmed.2020.10.044. 33316254 PMC7732230

[67] Kampouri E , Filippidis P , Viala B , et al. Predicting venous thromboembolic events in patients with coronavirus disease 2019 requiring hospitalization: an observational retrospective study by the COVIDIC initiative in a Swiss university hospital. Biomed Res Int 2020; 2020:9126148. 10.1155/2020/9126148. 33204727 PMC7656238

[68] Rukmana A , Sofiani Y , Agung RN . Efektivitas Skor Wells dengan Pemeriksaan D‐Dimer, Prothrombin Time, Activated Partial Thromboplastin Time dan Fibrinogen Terhadap Deteksi Dini Deep Vein Trhombosis di Ruang ICU RSPAD Gatot Soebroto. DK 2023; 11:324–337. 10.20527/jdk.v11i3.551.

[69] Zhang S , Li L , Shen A , et al. Rational use of tocilizumab in the treatment of novel coronavirus pneumonia. Clin Drug Investig 2020; 40:511–518. 10.1007/s40261-020-00917-3. PMC7183818 32337664

[70] Zhang X , Zhang Y , Qiao W , et al. Baricitinib, a drug with potential effect to prevent SARS‐COV‐2 from entering target cells and control cytokine storm induced by COVID‐19. Int Immunopharmacol 2020; 86:106749. 10.1016/j.intimp.2020.106749. 32645632 PMC7328558

[71] de Jong MD , Simmons CP , Thanh TT , et al. Fatal outcome of human influenza a (H5N1) is associated with high viral load and hypercytokinemia. Nat Med 2006; 12:1203–1207. 10.1038/nm1477. 16964257 PMC4333202

[72] Russell CD , Millar JE , Baillie JK . Clinical evidence does not support corticosteroid treatment for 2019‐nCoV lung injury. Lancet 2020; 395:473–475. 10.1016/S0140-6736(20)30317-2. 32043983 PMC7134694

[73] Ministério da Saúde . Diretrizes Brasileiras para Tratamento Hospitalar do Paciente com COVID‐19 2021.

[74] Hanff TC , Mohareb AM , Giri J , et al. Thrombosis in COVID‐19. Am J Hematol 2020; 95:1578–1589. 10.1002/ajh.25982. 32857878 PMC7674272

[75] Brasil M da S Instituto Brasileiro de Geografia e Estatística, Coordenação de Trabalho e Rendimento . Protocolo de manejo clínico da Covid‐19 na Atenção Especializada [recurso eletrônico]. Brasilia. 2020.

[76] Russo V , Di Maio M , Attena E , et al. Clinical impact of pre‐admission antithrombotic therapy in hospitalized patients with COVID‐19: a multicenter observational study. Pharmacol Res 2020; 159:104965. 10.1016/j.phrs.2020.104965. 32474087 PMC7256617

[77] Moores LK , Tritschler T , Brosnahan S , et al. Prevention, diagnosis, and treatment of VTE in patients with coronavirus disease 2019. Chest 2020; 158:1143–1163. 10.1016/j.chest.2020.05.559. 32502594 PMC7265858

[78] Susen S , Tacquard CA , Godon A , et al. Prevention of thrombotic risk in hospitalized patients with COVID‐19 and hemostasis monitoring. Crit Care 2020; 24:364. 10.1186/s13054-020-03000-7. 32560658 PMC7303590

