# Supplementary material for: Deep Vein Thrombosis in Critically Ill Patients With COVID‐19 Pneumonia: Incidence, Wells Score Diagnostic Performance, and Hospital Prognosis
Source: J Ultrasound Med. 2025 Sep 10;45(1):155–70. doi: 10.1002/jum.70046 (PMC12669431; doi:10.1002/jum.70046)
Supplement: Supplementary file 1 — Data S1. Supporting information. Table S1. Lung images baseline data by deep venous thrombosis diagnosis. Table S2. SIC score diagnostic performance in DVT among COVID‐19 patients. Table S3. SAPS 3 score diagnostic performance in DVT among COVID‐19 patients. Table S4. Charlson comorbidity index diagnostic performance in DVT among COVID‐19 patients. Table S5. SOFA score diagnostic performance in DVT among COVID‐19 patients. Table S6. Least square linear model DVT and SAPS 3 coefficients predicting log length of stay among Covid‐19 patients. Table S7. Cox proportional hazards model coefficients in hazard ratios of DVT for hemodialysis adjusted for SAPS 3 among Covid‐19 patients. Table S8. Least square linear model DVT and SAPS 3 coefficients predicting log length of mechanical ventilation among Covid‐19 patients. [file JUM-45-155-s001.docx]

**Table S1:** Lung images baseline data by deep venous thrombosis diagnosis.

|  | **Deep Venous Thrombosis** | |  |
| --- | --- | --- | --- |
|  | **No (N=168)** | **Yes (N=18)** | **Overall (N=186)** |
| **CXR Normal** |  |  |  |
| No | 168 (100%) | 18 (100%) | 186 (100%) |
| Yes | 0 (0%) | 0 (0%) | 0 (0%) |
| **CXR Consolidation** |  |  |  |
| No | 159 (94.6%) | 14 (77.8%) | 173 (93.0%) |
| Yes | 9 (5.4%) | 4 (22.2%) | 13 (7.0%) |
| **CXR Peripheral Lung Interstitial Infiltrates** |  |  |  |
| No | 159 (94.6%) | 15 (83.3%) | 174 (93.5%) |
| Yes | 9 (5.4%) | 3 (16.7%) | 12 (6.5%) |
| **CXR Diffuse Interstitial Lung Disease** |  |  |  |
| No | 148 (88.1%) | 15 (83.3%) | 163 (87.6%) |
| Yes | 20 (11.9%) | 3 (16.7%) | 23 (12.4%) |
| **CXR Pleural Effusion** |  |  |  |
| No | 157 (93.5%) | 16 (88.9%) | 173 (93.0%) |
| Yes | 11 (6.5%) | 2 (11.1%) | 13 (7.0%) |
| **CXR Others** |  |  |  |
| No | 152 (90.5%) | 16 (88.9%) | 168 (90.3%) |
| Yes | 16 (9.5%) | 2 (11.1%) | 18 (9.7%) |
| **CT Normal** |  |  |  |
| No | 167 (99.4%) | 18 (100%) | 185 (99.5%) |
| Yes | 1 (0.6%) | 0 (0%) | 1 (0.5%) |
| **CT Consolidation** |  |  |  |
| No | 84 (50.0%) | 9 (50.0%) | 93 (50.0%) |
| Yes | 84 (50.0%) | 9 (50.0%) | 93 (50.0%) |
| **CT Peripheral Lung Interstitial Infiltrates** |  |  |  |
| No | 161 (95.8%) | 18 (100%) | 179 (96.2%) |
| Yes | 7 (4.2%) | 0 (0%) | 7 (3.8%) |
| **CT Diffuse Interstitial Lung Disease** |  |  |  |
| No | 161 (95.8%) | 18 (100%) | 179 (96.2%) |
| Yes | 7 (4.2%) | 0 (0%) | 7 (3.8%) |
| **CT Ground-Glass** |  |  |  |
| No | 60 (35.7%) | 6 (33.3%) | 66 (35.5%) |
| Yes | 108 (64.3%) | 12 (66.7%) | 120 (64.5%) |
| **CT Pleural Effusion** |  |  |  |
| No | 136 (81.0%) | 14 (77.8%) | 150 (80.6%) |
| Yes | 32 (19.0%) | 4 (22.2%) | 36 (19.4%)^[[1]](#footnote-1)^ |
| **CT Others** |  |  |  |
| No | 127 (75.6%) | 8 (44.4%) | 135 (72.6%) |
| Yes | 41 (24.4%) | 10 (55.6%) | 51 (27.4%) |

CT= computed tomography; CXR = chest X-ray; Max = Maximum; Min = Minimum; SD = Standard Deviation;

**Table** **S2**: SIC score diagnostic performance in DVT among COVID-19 patients.

| **SIC Score** | **D** | **ND** | **TP** | **FN** | **FP** | **TN** | **Sensitivity** | **Se.inf.cl** | **Se.sup.cl** | **Specificity** | **Sp.inf.cl** | **Sp.sup.cl** |
| --- | --- | --- | --- | --- | --- | --- | --- | --- | --- | --- | --- | --- |
| 0 | 0 | 23 | 18 | 0 | 168 | 1 | **1.000** | 0.824 | 1.000 | **0.006** | 0.001 | 0.033 |
| 1 | 1 | 24 | 18 | 0 | 145 | 23 | **1.000** | 0.824 | 1.000 | **0.137** | 0.093 | 0.197 |
| 2 | 10 | 99 | 17 | 1 | 121 | 47 | **0.944** | 0.742 | 0.990 | **0.280** | 0.217 | 0.352 |
| 3 | 3 | 20 | 7 | 11 | 22 | 146 | **0.389** | 0.203 | 0.614 | **0.869** | 0.810 | 0.912 |
| 4 | 4 | 2 | 4 | 14 | 2 | 166 | **0.222** | 0.090 | 0.452 | **0.988** | 0.958 | 0.997 |
| cl=95% confidence limit; D=With Deep Vein Thrombosis; FN=False Negative; FP=False Positive; ND=Without Deep Vein Thrombosis; Se=Sensitivity; Sp=Specificity; TN=True Negative; TP=True Positive. The cut-off considered = 3 (estimation method is the maximization of the Youden J index). ROC AUC = 0.70 | | | | | | | | | | | | |

**Table** **S3**: SAPS 3 score diagnostic performance in DVT among COVID-19 patients.

| **SAPS3** | **D** | **ND** | **TP** | **FN** | **FP** | **TN** | **Sensitivity** | **Se.inf.cl** | **Se.sup.cl** | **Specificity** | **Sp.inf.cl** | **Sp.sup.cl** |
| --- | --- | --- | --- | --- | --- | --- | --- | --- | --- | --- | --- | --- |
| 18 | 0 | 3 | 18 | 0 | 168 | 1 | **1.000** | 0.824 | 1.000 | **0.006** | 0.001 | 0.033 |
| 20 | 0 | 1 | 18 | 0 | 165 | 3 | **1.000** | 0.824 | 1.000 | **0.018** | 0.006 | 0.051 |
| 21 | 0 | 3 | 18 | 0 | 164 | 4 | **1.000** | 0.824 | 1.000 | **0.024** | 0.009 | 0.060 |
| 22 | 0 | 1 | 18 | 0 | 161 | 7 | **1.000** | 0.824 | 1.000 | **0.042** | 0.020 | 0.083 |
| 23 | 0 | 4 | 18 | 0 | 160 | 8 | **1.000** | 0.824 | 1.000 | **0.048** | 0.024 | 0.091 |
| 24 | 0 | 5 | 18 | 0 | 156 | 12 | **1.000** | 0.824 | 1.000 | **0.071** | 0.041 | 0.121 |
| 26 | 0 | 3 | 18 | 0 | 151 | 17 | **1.000** | 0.824 | 1.000 | **0.101** | 0.064 | 0.156 |
| 28 | 0 | 2 | 18 | 0 | 148 | 20 | **1.000** | 0.824 | 1.000 | **0.119** | 0.078 | 0.177 |
| 31 | 0 | 1 | 18 | 0 | 146 | 22 | **1.000** | 0.824 | 1.000 | **0.131** | 0.088 | 0.190 |
| 32 | 0 | 4 | 18 | 0 | 145 | 23 | **1.000** | 0.824 | 1.000 | **0.137** | 0.093 | 0.197 |
| 33 | 0 | 7 | 18 | 0 | 141 | 27 | **1.000** | 0.824 | 1.000 | **0.161** | 0.113 | 0.224 |
| 34 | 0 | 6 | 18 | 0 | 134 | 34 | **1.000** | 0.824 | 1.000 | **0.202** | 0.149 | 0.269 |
| 35 | 0 | 2 | 18 | 0 | 128 | 40 | **1.000** | 0.824 | 1.000 | **0.238** | 0.180 | 0.308 |
| 36 | 0 | 9 | 18 | 0 | 126 | 42 | **1.000** | 0.824 | 1.000 | **0.250** | 0.191 | 0.321 |
| 37 | 0 | 1 | 18 | 0 | 117 | 51 | **1.000** | 0.824 | 1.000 | **0.304** | 0.239 | 0.377 |
| 38 | 0 | 10 | 18 | 0 | 116 | 52 | **1.000** | 0.824 | 1.000 | **0.310** | 0.245 | 0.383 |
| 39 | 0 | 1 | 18 | 0 | 106 | 62 | **1.000** | 0.824 | 1.000 | **0.369** | 0.300 | 0.444 |
| 40 | 1 | 1 | 18 | 0 | 105 | 63 | **1.000** | 0.824 | 1.000 | **0.375** | 0.305 | 0.450 |
| 41 | 0 | 5 | 17 | 1 | 104 | 64 | **0.944** | 0.742 | 0.990 | **0.381** | 0.311 | 0.456 |
| 42 | 1 | 3 | 17 | 1 | 99 | 69 | **0.944** | 0.742 | 0.990 | **0.411** | 0.339 | 0.486 |
| 43 | 1 | 5 | 16 | 2 | 96 | 72 | **0.889** | 0.672 | 0.969 | **0.429** | 0.356 | 0.504 |
| 44 | 1 | 8 | 15 | 3 | 91 | 77 | **0.833** | 0.608 | 0.942 | **0.458** | 0.385 | 0.534 |
| 45 | 1 | 2 | 14 | 4 | 83 | 85 | **0.778** | 0.548 | 0.910 | **0.506** | 0.431 | 0.581 |
| 46 | 1 | 3 | 13 | 5 | 81 | 87 | **0.722** | 0.491 | 0.875 | **0.518** | 0.443 | 0.592 |
| 47 | 0 | 2 | 12 | 6 | 78 | 90 | **0.667** | 0.437 | 0.837 | **0.536** | 0.460 | 0.609 |
| 48 | 0 | 7 | 12 | 6 | 76 | 92 | **0.667** | 0.437 | 0.837 | **0.548** | 0.472 | 0.621 |
| 49 | 0 | 2 | 12 | 6 | 69 | 99 | **0.667** | 0.437 | 0.837 | **0.589** | 0.514 | 0.661 |
| 50 | 0 | 1 | 12 | 6 | 67 | 101 | **0.667** | 0.437 | 0.837 | **0.601** | 0.526 | 0.672 |
| 52 | 0 | 1 | 12 | 6 | 66 | 102 | **0.667** | 0.437 | 0.837 | **0.607** | 0.532 | 0.678 |
| 53 | 0 | 2 | 12 | 6 | 65 | 103 | **0.667** | 0.437 | 0.837 | **0.613** | 0.538 | 0.683 |
| 54 | 0 | 3 | 12 | 6 | 63 | 105 | **0.667** | 0.437 | 0.837 | **0.625** | 0.550 | 0.695 |
| 55 | 0 | 1 | 12 | 6 | 60 | 108 | **0.667** | 0.437 | 0.837 | **0.643** | 0.568 | 0.711 |
| 56 | 1 | 7 | 12 | 6 | 59 | 109 | **0.667** | 0.437 | 0.837 | **0.649** | 0.574 | 0.717 |
| 57 | 2 | 0 | 11 | 7 | 52 | 116 | **0.611** | 0.386 | 0.797 | **0.690** | 0.617 | 0.755 |
| 58 | 0 | 3 | 9 | 9 | 52 | 116 | **0.500** | 0.290 | 0.710 | **0.690** | 0.617 | 0.755 |
| 59 | 0 | 1 | 9 | 9 | 49 | 119 | **0.500** | 0.290 | 0.710 | **0.708** | 0.636 | 0.772 |
| 62 | 2 | 2 | 9 | 9 | 48 | 120 | **0.500** | 0.290 | 0.710 | **0.714** | 0.642 | 0.777 |
| 63 | 0 | 3 | 7 | 11 | 46 | 122 | **0.389** | 0.203 | 0.614 | **0.726** | 0.654 | 0.788 |
| 64 | 0 | 2 | 7 | 11 | 43 | 125 | **0.389** | 0.203 | 0.614 | **0.744** | 0.673 | 0.804 |
| 65 | 0 | 4 | 7 | 11 | 41 | 127 | **0.389** | 0.203 | 0.614 | **0.756** | 0.686 | 0.815 |
| 66 | 1 | 2 | 7 | 11 | 37 | 131 | **0.389** | 0.203 | 0.614 | **0.780** | 0.711 | 0.836 |
| 67 | 0 | 2 | 6 | 12 | 35 | 133 | **0.333** | 0.163 | 0.563 | **0.792** | 0.724 | 0.846 |
| 68 | 1 | 3 | 6 | 12 | 33 | 135 | **0.333** | 0.163 | 0.563 | **0.804** | 0.737 | 0.857 |
| 69 | 0 | 1 | 5 | 13 | 30 | 138 | **0.278** | 0.125 | 0.509 | **0.821** | 0.757 | 0.872 |
| 71 | 0 | 1 | 5 | 13 | 29 | 139 | **0.278** | 0.125 | 0.509 | **0.827** | 0.763 | 0.877 |
| 72 | 0 | 1 | 5 | 13 | 28 | 140 | **0.278** | 0.125 | 0.509 | **0.833** | 0.770 | 0.882 |
| 73 | 0 | 4 | 5 | 13 | 27 | 141 | **0.278** | 0.125 | 0.509 | **0.839** | 0.776 | 0.887 |
| 74 | 0 | 3 | 5 | 13 | 23 | 145 | **0.278** | 0.125 | 0.509 | **0.863** | 0.803 | 0.907 |
| 75 | 0 | 1 | 5 | 13 | 20 | 148 | **0.278** | 0.125 | 0.509 | **0.881** | 0.823 | 0.922 |
| 76 | 0 | 4 | 5 | 13 | 19 | 149 | **0.278** | 0.125 | 0.509 | **0.887** | 0.830 | 0.926 |
| 78 | 3 | 3 | 5 | 13 | 15 | 153 | **0.278** | 0.125 | 0.509 | **0.911** | 0.858 | 0.945 |
| 79 | 1 | 0 | 2 | 16 | 12 | 156 | **0.111** | 0.031 | 0.328 | **0.929** | 0.879 | 0.959 |
| 80 | 0 | 2 | 1 | 17 | 12 | 156 | **0.056** | 0.010 | 0.258 | **0.929** | 0.879 | 0.959 |
| 81 | 0 | 1 | 1 | 17 | 10 | 158 | **0.056** | 0.010 | 0.258 | **0.940** | 0.894 | 0.967 |
| 82 | 1 | 6 | 1 | 17 | 9 | 159 | **0.056** | 0.010 | 0.258 | **0.946** | 0.901 | 0.972 |
| 83 | 0 | 1 | 0 | 18 | 3 | 165 | **0.000** | 0.000 | 0.176 | **0.982** | 0.949 | 0.994 |
| 88 | 0 | 2 | 0 | 18 | 2 | 166 | **0.000** | 0.000 | 0.176 | **0.988** | 0.958 | 0.997 |
| cl=95% confidence limit; D=With Deep Vein Thrombosis; FN=False Negative; FP=False Positive; ND=Without Deep Vein Thrombosis; Se=Sensitivity; Sp=Specificity; TN=True Negative; TP=True Positive; The cut-off considered 40 (estimation method is the maximization of the Youden J index). ROC AUC = 0.69 | | | | | | | | | | | | |

**Table** **S4**: Charlson comorbidity index diagnostic performance in DVT among COVID-19 patients.

| **Charlson Score** | **D** | **ND** | **TP** | **FN** | **FP** | **TN** | **Sensitivity** | **Se.inf.cl** | **Se.sup.cl** | **Specificity** | **Sp.inf.cl** | **Sp.sup.cl** |
| --- | --- | --- | --- | --- | --- | --- | --- | --- | --- | --- | --- | --- |
| 0 | 0 | 24 | 18 | 0 | 168 | 1 | **1.000** | 0.824 | 1.000 | **0.006** | 0.001 | 0.033 |
| 1 | 3 | 17 | 18 | 0 | 144 | 24 | **1.000** | 0.824 | 1.000 | **0.143** | 0.098 | 0.204 |
| 2 | 2 | 38 | 15 | 3 | 127 | 41 | **0.833** | 0.608 | 0.942 | **0.244** | 0.185 | 0.314 |
| 3 | 3 | 24 | 13 | 5 | 89 | 79 | **0.722** | 0.491 | 0.875 | **0.470** | 0.396 | 0.546 |
| 4 | 4 | 21 | 10 | 8 | 65 | 103 | **0.556** | 0.337 | 0.754 | **0.613** | 0.538 | 0.683 |
| 5 | 3 | 23 | 6 | 12 | 44 | 124 | **0.333** | 0.163 | 0.563 | **0.738** | 0.667 | 0.799 |
| 6 | 0 | 18 | 3 | 15 | 21 | 147 | **0.167** | 0.058 | 0.392 | **0.875** | 0.816 | 0.917 |
| 8 | 3 | 2 | 3 | 15 | 3 | 165 | **0.167** | 0.058 | 0.392 | **0.982** | 0.949 | 0.994 |
| 10 | 0 | 1 | 0 | 18 | 1 | 167 | **0.000** | 0.000 | 0.176 | **0.994** | 0.967 | 0.999 |
| cl=95% confidence limit; D=With Deep Vein Thrombosis; FN=False Negative; FP=False Positive; ND=Without Deep Vein Thrombosis; Se=Sensitivity; Sp=Specificity; TN=True Negative; TP=True Positive. The cut-off considered 3 (estimation method is the maximization of the Youden J index), ROC AUC = 0.61  . | | | | | | | | | | | | |

**Table** **S5**: SOFA score diagnostic performance in DVT among COVID-19 patients.

| **SOFA Score** | **D** | **ND** | **TP** | **FN** | **FP** | **TN** | **Sensitivity** | **Se.inf.cl** | **Se.sup.cl** | **Specificity** | **Sp.inf.cl** | **Sp.sup.cl** |
| --- | --- | --- | --- | --- | --- | --- | --- | --- | --- | --- | --- | --- |
| 0 | 0 | 23 | 18 | 0 | 168 | 1 | **1.000** | 0.824 | 1.000 | **0.006** | 0.001 | 0.033 |
| 1 | 1 | 25 | 18 | 0 | 145 | 23 | **1.000** | 0.824 | 1.000 | **0.137** | 0.093 | 0.197 |
| 2 | 3 | 32 | 17 | 1 | 120 | 48 | **0.944** | 0.742 | 0.990 | **0.286** | 0.223 | 0.358 |
| 3 | 0 | 18 | 14 | 4 | 88 | 80 | **0.778** | 0.548 | 0.910 | **0.476** | 0.402 | 0.551 |
| 4 | 1 | 9 | 14 | 4 | 70 | 98 | **0.778** | 0.548 | 0.910 | **0.583** | 0.508 | 0.655 |
| 5 | 1 | 5 | 13 | 5 | 61 | 107 | **0.722** | 0.491 | 0.875 | **0.637** | 0.562 | 0.706 |
| 6 | 1 | 6 | 12 | 6 | 56 | 112 | **0.667** | 0.437 | 0.837 | **0.667** | 0.592 | 0.734 |
| 7 | 1 | 4 | 11 | 7 | 50 | 118 | **0.611** | 0.386 | 0.797 | **0.702** | 0.629 | 0.766 |
| 8 | 3 | 9 | 10 | 8 | 46 | 122 | **0.556** | 0.337 | 0.754 | **0.726** | 0.654 | 0.788 |
| 9 | 1 | 17 | 7 | 11 | 37 | 131 | **0.389** | 0.203 | 0.614 | **0.780** | 0.711 | 0.836 |
| 10 | 4 | 8 | 6 | 12 | 20 | 148 | **0.333** | 0.163 | 0.563 | **0.881** | 0.823 | 0.922 |
| 11 | 1 | 3 | 2 | 16 | 12 | 156 | **0.111** | 0.031 | 0.328 | **0.929** | 0.879 | 0.959 |
| 12 | 1 | 8 | 1 | 17 | 9 | 159 | **0.056** | 0.010 | 0.258 | **0.946** | 0.901 | 0.972 |
| 13 | 0 | 1 | 0 | 18 | 1 | 167 | **0.000** | 0.000 | 0.176 | **0.994** | 0.967 | 0.999 |
| cl=95% confidence limit; D=With Deep Vein Thrombosis; FN=False Negative; FP=False Positive; ND=Without Deep Vein Thrombosis; Se=Sensitivity; Sp=Specificity; TN=True Negative; TP=True Positive. The cut-off considered 4 (estimation method is the maximization of the Youden J index), ROC AUC = 0.70 | | | | | | | | | | | | |

**Table** **S6**: Least square linear model DVT and SAPS 3 coefficients predicting log length of stay among Covid-19 patients.

| **Variables** | **Effect** | **S.E.** | **Lower 0.95** | **Upper 0.95** |
| --- | --- | --- | --- | --- |
| SAPS 3 | 0.312 | 0.081 | 0.153 | 0.471 |
| DVT - Yes:No | 0.059 | 0.170 | -0.276 | 0.395 |
| Lower = confidence limit; Upper = confidence limit; S.E. = standard error; R2 adjusted: 0.071 | | | | |

**Table S7**: Cox proportional hazards model coefficients in hazard ratios of DVT for hemodialysis adjusted for SAPS 3 among Covid-19 patients.

| **Variables** | **Effect** | **S.E.** | **Lower 0.95** | **Upper 0.95** |
| --- | --- | --- | --- | --- |
| SAPS 3 | 2.003 | 0.369 | 1.281 | 2.726 |
| DVT - Yes:No | -0.032 | 0.538 | -1.086 | 1.022 |
| Lower = confidence limit; Upper = confidence limit; S.E. = standard error; R2:0.226 | | | | |

**Table** **S8**: Least square linear model DVT and SAPS 3 coefficients predicting log length of mechanical ventilation among Covid-19 patients.

| **Variables** | **Effect** | **S.E.** | **Lower 0.95** | **Upper 0.95** |
| --- | --- | --- | --- | --- |
| SAPS 3 | 0.346 | 0.133 | 0.083 | 0.610 |
| DVT - Yes:No | -0.445 | 0.231 | -0.904 | 0.015 |
| Lower = confidence limit; Upper = confidence limit; S.E. = standard error; R2 adjusted: 0.075 | | | | |

1. Continued next page [↑](#footnote-ref-1)
